# Pain expressiveness and altruistic behavior: an exploration using agent-based modeling

**DOI:** 10.1097/j.pain.0000000000000443

**Published:** 2015-11-26

**Authors:** Amanda C. de C Williams, Elizabeth Gallagher, Antonio R. Fidalgo, Peter J. Bentley

**Affiliations:** a Research Department of Clinical, Educational & Health Psychology, University College London, London, United Kingdom; b Centre for Mathematics and Physics in the Life Sciences & Experimental Biology (CoMPLEX), University College London, London, United Kingdom; c ULC Interaction Centre, University College London, London, United Kingdom; d Department of Computer Science, University College London, London, United Kingdom

**Keywords:** Evolution, Helping, Exploitation, Artificial life

## Abstract

Predictions which invoke evolutionary mechanisms are hard to test. Agent-based modeling in artificial life offers a way to simulate behaviors and interactions in specific physical or social environments over many generations. The outcomes have implications for understanding adaptive value of behaviors in context. Pain-related behavior in animals is communicated to other animals that might protect or help, or might exploit or predate. An agent-based model simulated the effects of displaying or not displaying pain (expresser/nonexpresser strategies) when injured and of helping, ignoring, or exploiting another in pain (altruistic/nonaltruistic/selfish strategies). Agents modeled in MATLAB interacted at random while foraging (gaining energy); random injury interrupted foraging for a fixed time unless help from an altruistic agent, who paid an energy cost, speeded recovery. Environmental and social conditions also varied, and each model ran for 10,000 iterations. Findings were meaningful in that, in general, contingencies that evident from experimental work with a variety of mammals, over a few interactions, were replicated in the agent-based model after selection pressure over many generations. More energy-demanding expression of pain reduced its frequency in successive generations, and increasing injury frequency resulted in fewer expressers and altruists. Allowing exploitation of injured agents decreased expression of pain to near zero, but altruists remained. Decreasing costs or increasing benefits of helping hardly changed its frequency, whereas increasing interaction rate between injured agents and helpers diminished the benefits to both. Agent-based modeling allows simulation of complex behaviors and environmental pressures over evolutionary time.

## Abstract 2

Supplemental Digital Content is Available in the Text.

Agent-based modeling is a widely applicable in silico method. In this study, it showed pain expression and helping others to be evolutionarily viable behaviors under some conditions.

## 1. Introduction

Injury represents a major threat to animals' survival and fitness, and pain serves to prioritize efforts to escape and to promote recovery.^52,57^ Thus behavior associated with pain in animals is of particular interest, but the evolutionary perspective has been neglected.^55,57^

Testing evolutionary explanations is difficult, whereas fitting speculative explanations to observational data is unsatisfactory.^24^ Computer simulation of the effects of selection pressures on behaviors over generations, using agent-based models and in silico experiments,^2^ offers a viable alternative.^3,38^ Whole system dynamics emerge from the interactions of agents that (1) are discrete and self-contained with characteristics and rules for behavior, (2) interact with other agents in an environment to which they can respond, (3) are capable of functioning independently of the environment and of other agents, and (4) have goals and may change behaviors based on experience.^20,32^

We applied this method to the expression of pain and possible responses by conspecifics, in a generic simplified mammal-like model. Pain provides an immediate signal of threat or actual harm and subsequently prompts wound care^52^ and protective behaviors,^13,33,34^ which in turn alert conspecifics to proximal danger. Responses by conspecifics would have coevolved with the expression of pain,^55^ and some animals in some contexts show prosocial behavior toward the affected individual. Consistencies in behavior associated with acute pain have been noted in many invertebrates,^53^ and in all classes of vertebrates,^45,47^ while responses to others' pain have been studied in rodents^5,16,29,36^ and in humans.^51,55^ Prosocial behavior in animals is explained by shared genes,^50,58^ or by reciprocal exchange,^26,50^ supported by psychological adaptations, with nonreciprocators recognized and excluded.^12,17,22,26,49^ These adaptations foster commitment in close social groups^14,22,49^ and reputation in a wider group.^21,40,48^

All behavior has energy costs that are offset against goals. Pain behavior also signals vulnerability, and conspecifics may take advantage, stealing food, or usurping social position, or predators may target the individual.^35^ Thus pain expression varies with social context,^56^ as shown in mice by Mogil et al.^28,30^ Humans in the presence of social threat suppress facial expression of pain^42^ while rating their pain as worse than without threat,^54^ whereas support from another mitigates pain experience^9^ and pain-related brain activity.^11^

Therefore, the contingencies under which expressing pain and helping those in pain might increase or decline over evolutionary time can be investigated by agent-based modeling in a population with mixed behaviors and under different environmental conditions. The parameters used were displaying or not displaying pain, and a prosocial response or no response or an exploitative response. So, in our simulation, agents either expressed or suppressed pain when (randomly) injured, and helped, ignored, or exploited others in pain. Agents suffered or benefited, or neither, from these interactions, and this affected their fitness and the balance of behaviors in the population after many generations.

## 2. Methods

**Figure 1:**
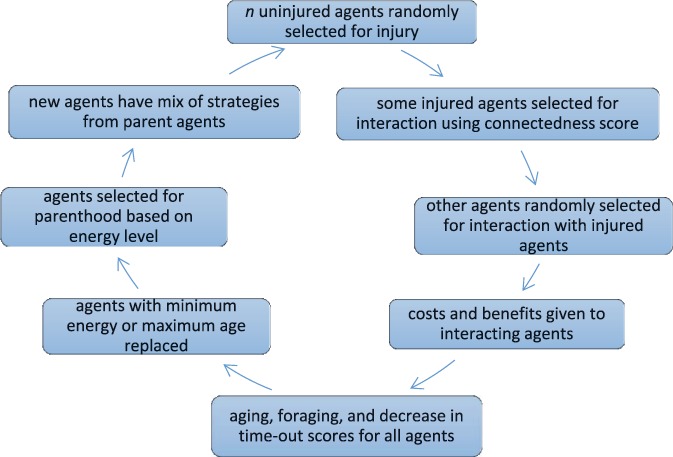
Processes in the model.

The agent-based model is shown in Figure 1. The agent is envisaged as a generic mammal with the capacity to show pain,^37^ to provide help to another agent in pain,^5,16,36^ and with social tendencies (though some nonmammalian vertebrates also show similar behaviors^13,47,53^). Pain-related behavior and the responses of others occurred in the context of a finite life spent foraging to gain energy, such that injury prevented foraging, and help enabled an earlier return to foraging. We also introduced variability in sociability of agents, not unlike dimensions of shyness or boldness described, for instance, in some fish and cephalopods.^1,6,10,13^

We used these variables to address the following questions: (1) What was the effect of increasing the energy costs of expressing pain? (2) What was the effect of increasing or decreasing the costs of helping an agent in pain? (3) What was the effect of increasing agents' sociability by increasing interaction rate? (4) What was the effect of exploitation by healthy agents of injured agents that expressed pain? (5) What is the effect of increasing the frequency of injury on expression of pain? (6) What is the effect of increasing recovery time from injury on expression of pain?

MATLAB is a computing environment and full programming language, which offers the ability to model complex relationships for simulation and to manage data from the simulation, allowing detailed analysis. To demonstrate the method, we manipulated 1 variable at a time to address each question. In each run of the model, 100 agents foraged (gained energy), but some selected at random were injured and had pain, preventing foraging for a fixed interval. Agents interacted so that an expressive agent in pain meeting an altruistic agent received help and could return to foraging, whereas the altruistic agent providing help thereby lost energy. The nonexpressive agent in pain, by virtue of not communicating need, could not receive help. The nonaltruistic agent gave no help and so lost no energy in interaction with an expressive agent in pain. Agents died when their energy was spent or they were old. Dead agents were replaced by new agents bearing the characteristics of the highest energy surviving agents, effectively their parents. The MATLAB code can be found in online Supplemental Data 1 (available online as Supplemental Digital Content at http://links.lww.com/PAIN/A191). Details of the model follow:

Each agent had an age, an energy level (equivalent to fitness), an expression strategy (express or suppress pain when injured), an altruism strategy (help or ignore an agent expressing pain), a connectedness score (likelihood of interaction), and a recovery time score. The 2 strategies (expression or suppression; help or ignore) were stable across the agent's lifetime, but energy level and age changed. In addition, the environment determined the frequency of injury and changes in recovery time from injury and in interaction rate.

### 2.1. Agent properties

#### 2.1.1. Age

Agents lived for a set number of iterations, gaining a value of 1 each iteration up to a maximum of 100 at which point they died and were replaced. Initial ages were taken from a random uniform distribution between 1 and a maximum age.

#### 2.1.2. Energy

Initial energy (fitness) levels were the same across agents, 10 of a maximum 20, and at each iteration, uninjured agents foraged at a steady rate and thereby gained energy by increments of 1. Agents who reached the maximum energy level of 20 continued to forage, maintaining but not gaining energy. If energy fell to 0, the agent died. Parents of new agents to replace those who died were selected based on their energy levels (fitness). The offspring agent inherited the expression or suppression and helping or ignoring strategies from its parents, each strategy selected at random from 1 parent.

#### 2.1.3. Expressing/suppressing pain strategy

Agents had a stable strategy across the lifespan of either expressing pain when injured, such that it was visible to an interacting agent, or suppressing pain, so that it was not visible. The energy cost of expressing pain, c_exp_, varied from 0 to 20, representing behavior from a brief facial grimace to extended limping, guarding, and other gross motor behavior.

#### 2.1.4. Helping = “altruistic”/ignoring = “nonaltruistic” strategies

Agents had a stable strategy across the lifespan of either helping an injured agent who was expressing pain or ignoring an injured agent expressing pain. These were described as altruistic and nonaltruistic strategies. Neither strategy was used in response to an agent who suppressed pain behavior. Altruism had an energy cost, c_alt_, which varied from 0 to 20 to lie within the same range as the energy costs of expressing pain, although the are not necessarily symmetrical; nonaltruistic behavior had none.

Thus, agents could express pain and altruistically help others in pain, express pain and nonaltruistically ignore others in pain, not express pain but altruistically help others, or not express pain and nonaltruistically ignore others in pain.

#### 2.1.5. Connectedness

Agents had a connectedness index between 0.1 and 0.9, which determined the likelihood of interacting with other agents, randomly assigned at birth from an exponential distribution and shifted such that few agents had high connectedness and most had low values, consistent with findings in social networks.^14^ If an agent helped another agent in pain, connectedness of the helper increased by the benefit b_alt_ that varied from 0 to 1 with a default of 0.5. If the agent ignored another agent in pain, its connectedness decreased by the cost, c_self_, varied from 0 to 1 with a default of 0.5. This represented reputation, and for agents who expressed pain, increased connectedness was associated with increased likelihood of interaction with another agent, and thus, the possibility of receiving help. Conversely, agents who ignored others in pain decreased in connectedness and, therefore, when they were in pain and expressed it, they were less likely to interact with any agent, including helpers.

#### 2.1.6. Exploitation

The interacting healthy selfish agent was allowed to “steal” some energy from the injured agent; the amount of energy stolen was varied between 5 and 20 (at which point the injured agent would always die). The aim was to simulate exploitation of the injured agent in the form of predation, stealing resources, etc. At baseline, all agents had an exploitation energy score of 0.

### 2.2. Environmental variation

#### 2.2.1. Sociability

Social animals vary in their frequency of interaction or possibility of interaction by proximity. Higher levels of sociability imply greater proximity, thus, a greater chance of an individual in pain is being observed by others who might or might not help. The chance of interaction was increased by adding a constant s to the connectedness score, increasing the probability of all agents of interacting with others; s varied between 0 and 1 with a default value of 0.

#### 2.2.2. Time in pain

Injured agents were forced to take time out from foraging; the length of time, t_p_, varied from 0 to 100 with a default of 50. The only event which could shorten this recovery time was being helped by another agent; therefore, increased time in pain was effectively a cost of not expressing pain.

### 2.3. Interactions: injury, helping

#### 2.3.1. Injury/pain

At each iteration, a number of healthy agents were randomly selected for injury and thus pain. This imposed time out from foraging. Of these injured agents, and those from previous iterations who had not yet recovered, a certain number were selected, based on their connectedness score, to interact with randomly selected other agents. In different runs, the injury rate was varied from 1 in 100 to 10 in 100, representing environments of different dangerousness.

#### 2.3.2. Interaction

Agents were selected for interaction based on their connectedness score, without reference to whether 1 was injured and in pain. The outcomes of the interaction where 1 agent was injured and in pain depended on the expression/suppression strategy of that agent and on the helping/ignoring strategy of the other (if not injured). Table 1 shows the relative costs and benefits.

**Table 1:**
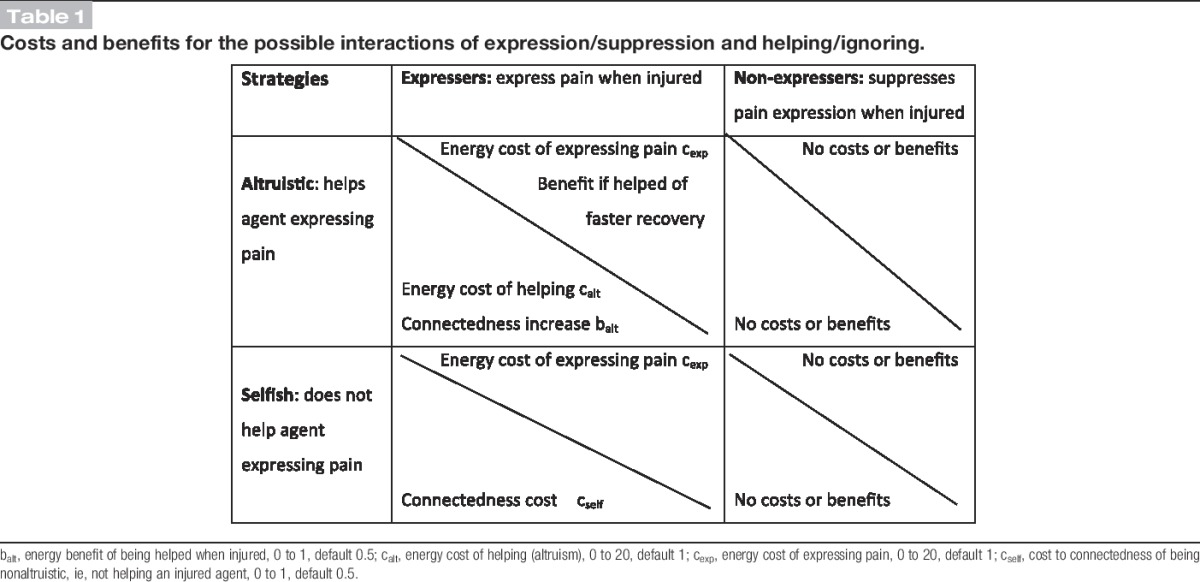
Costs and benefits for the possible interactions of expression/suppression and helping/ignoring.

The 5 cost and benefit parameters, cost of expression c_exp_, cost of altruistic behavior c_alt_, cost to connectedness of nonaltruistic behavior c_self_, benefit of altruistic behavior b_alt_, and time in pain t_p_, were calibrated using assumptions to produce the default values, as described in Supplemental Data 2 (available online as Supplemental Digital Content at http://links.lww.com/PAIN/A191).

### 2.4. Preliminary simulations

**Figure 2:**
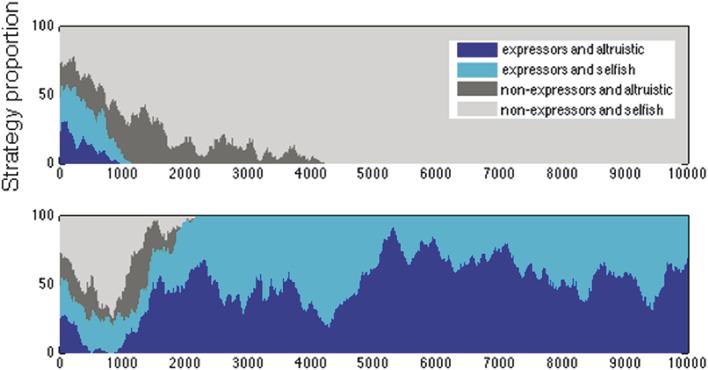
Single strategy dominance: 2 examples of simulations in which all agents end with a single strategy, after different numbers of iterations. See also Supplemental data 3 (available online as Supplemental Digital Content at http://links.lww.com/PAIN/A191).

**Figure 3:**
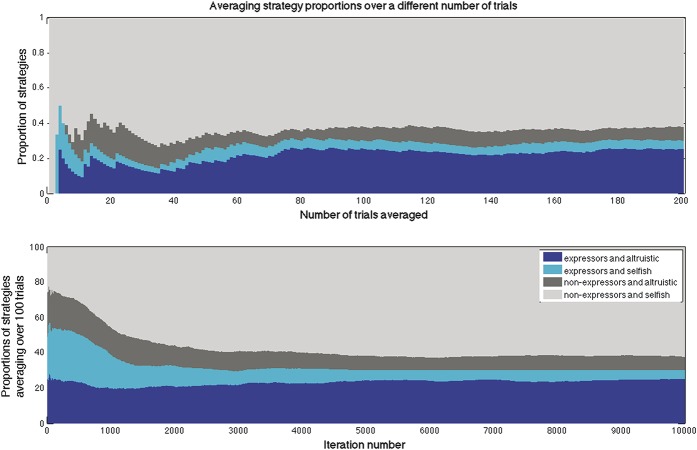
Determining default parameters: average final strategy proportions stay similar when averaging from at least 100 trials (above), and when averaged over 100 trials, 10,000 iterations achieve a stable balance of strategy proportions (below).

We considered the outcomes of 1 initial agent population, rather than many, examining the outcomes both of 10 repeated simulations of a single, default initial agent population, and of 10 different initial populations, using default parameters and averaging results over 100 repeated simulations run for 10,000 iterations. These preliminary simulations (Supplemental Data 2, available online as Supplemental Digital Content at http://links.lww.com/PAIN/A191) demonstrated the final proportions of strategies in the population in both conditions. Using the same default initial agent population and running 100 simulations with different random seeds, 60% of simulations ended in nonaltruistic nonexpresser strategies, 24% in altruistic expresser strategies, 6% as altruistic nonexpresser, and 4% as nonaltruistic expresser. The remaining 6% consisted of 4% with a mixture of nonexpresser strategies and 2% with a mixture of expresser strategies (Fig. 2 and Supplemental Data 2 for examples of individual simulations). After 10,000 iterations, almost all the trials would end in a single strategy outcome, and averaging beyond 100 repetitions did not yield any significant changes in the agents' strategies (Fig. 3). A 2-sample *t* test indicated that the data from both these sets of results had equal means and variances (with all *P* > 0.1). Hence, it is likely that most of the variance seen is due to the stochasticity of the model, rather than the stochasticity in finding the initial agents.

## 3. Results

### 3.1. Varying costs and benefits of interactions

To test the effects of varying costs and benefits of strategies, each parameter was varied, whereas others remained at their default values; as in the preliminary trials, for each variation, the proportion of each strategy at the 10,000th iteration is presented, averaged over 100 trials.

**Figure 4:**
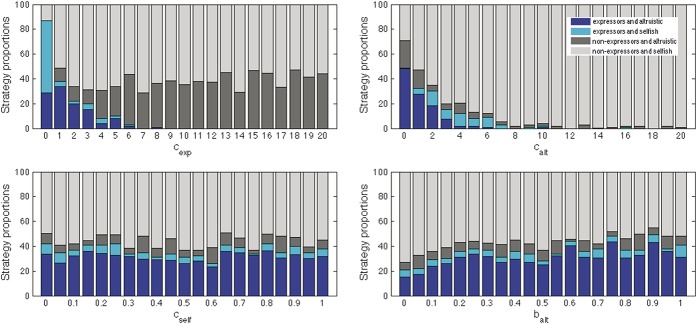
The effect of changing parameters on final strategy proportions: results from averaging 100 trials at the end of 10,000 iterations. (A, top left) Varying costs of expression affected the proportion of expressers. (B, top right) Varying costs of helping indirectly affected the proportions of expressers. (C, bottom left) Varying costs of not helping had little effect. (D, bottom right) Varying benefits of helping had little effect. Key: b_alt_, energy benefit of being helped when injured, 0 to 1, default 0.5; c_alt_, energy cost of helping (altruism), 0 to 20; c_exp_, energy cost of expressing pain, 0 to 20; c_self_, cost to connectedness of being nonaltruistic, ie, not helping an injured agent, 0 to 1, default 0.5.

(1) What was the effect of increasing the costs of expressing pain? As described in the Methods, costs of expressing pain c_exp_ were varied systematically to identify the effects on the balances of strategies. As the energy cost of expression increased, expresser strategies decreased to zero (Fig. 4A).
(2) What was the effect of increasing or decreasing the costs of helping agents in pain? Increasing the costs of altruism, that is, the energy costs of helping an injured agent, resulted in an indirect effect on the proportion of expressers, with even nonaltruistic expresser agents decreasing (Fig. 4B). Higher costs to altruists of helping were represented by lower energy scores and earlier death; the costs of expressing pain were less likely to be offset by a reasonable likelihood of being helped to recover, given the smaller numbers of altruists.

Varying the costs of being nonaltruistic, of not helping, made little difference to outcomes (Fig. 4C), with a predominance of nonaltruistic nonexpressers and altruistic expressers in relatively stable proportions. Similarly, varying the benefits of altruism (Fig. 4D) resulted in a small increase in altruistic expressers, but nonaltruistic nonexpressers remained in the majority.

**Figure 5:**
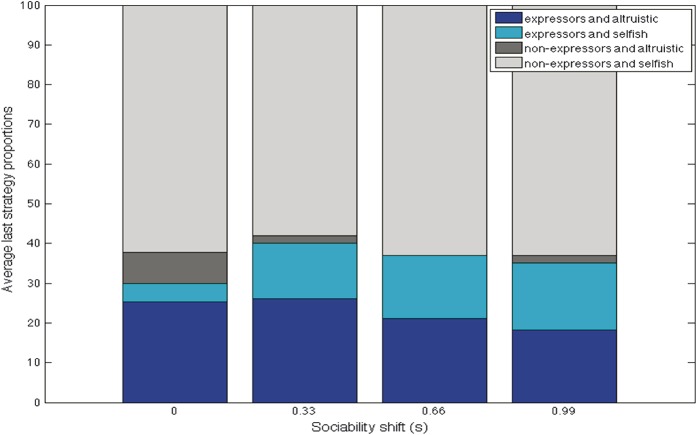
Increasing sociability s: the relative benefits of altruism seemed to decrease regardless of other strategies, averaged over 10,000 iterations and 100 trials. Key: s, increase in sociability, in addition to existing connectedness, 0 to 1.

**Figure 6:**
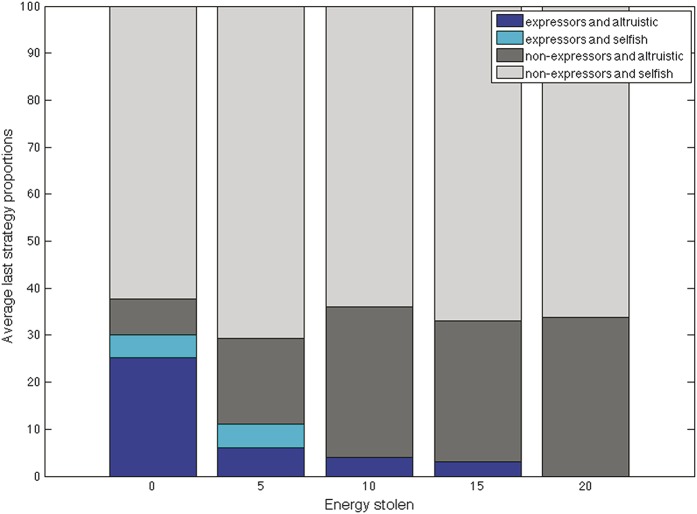
Exploiting the injured: when selfish agents could steal an injured agent's energy, expressing pain disappeared from the population, averaged over 10,000 iterations and 100 trials.

(3) What was the effect of varying agents' sociability? Manipulation of sociability by increasing the chances of interaction over the stable connectedness value of all agents had a small effect on the proportions of nonaltruistic expressers, which increased, and altruistic expressers, which decreased. The relative benefits of altruism to the altruistic and to the injured agent seemed to diminish as interactions increased, regardless of strategies (Fig. 5).
(4) What was the effect of allowing healthy agents to exploit injured agents? When we allowed selfish agents to steal energy from injured agents with which they interacted, a clear cost to expressing pain emerged (Fig. 6), proportional to the amount of energy stolen, with expressers disappearing from the population but altruists remaining.

### 3.2. Varying environmental parameters

**Figure 7:**
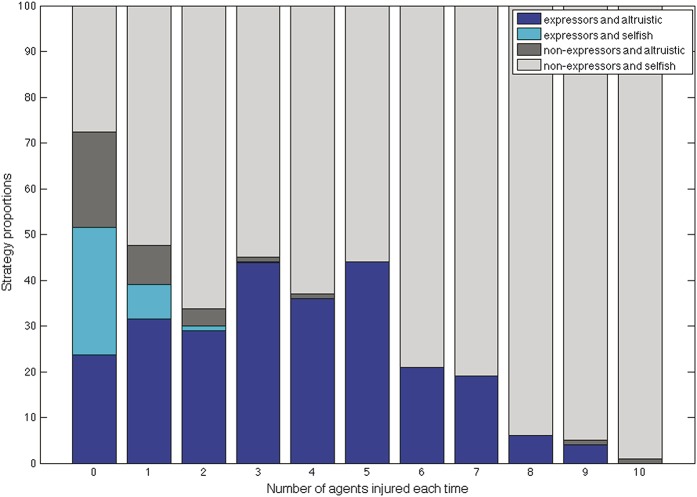
Increasing the frequency of injury: when n increases both expressing pain and altruism decrease, averaged over 10,000 iterations and 100 trials. When n is high (>8) almost all of the population is injured.

**Figure 8:**
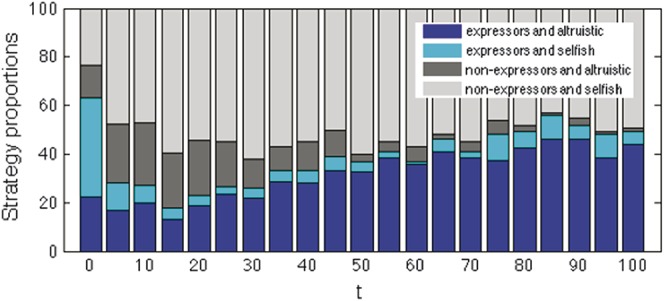
Varying time cost of injury: increased time to recover from injury resulted in a limited increase in expressers, averaged over 10,000 iterations and 100 trials.

(5) What was the effect of increasing the frequency of injury? Increasing the dangerousness of the environment, that is, the frequency (n) of injury, produced a decrease of expressers (Fig. 7), as the benefits of expressing injury and returning to foraging sooner were offset by high likelihood of reinjury, even during recovery. Helping also diminished rapidly as the environment became more dangerous, although it reemerged in very small proportions at higher levels.
(6) What was the effect of increasing recovery time from injury? When time in pain was increased (thereby making the cost of nonexpression higher), expresser strategies increased but only to a maximum of approximately 50% of the population (Fig. 8).

## 4. Discussion

Despite the limitations of a simplified and generic model, agent-based modeling provided an effective way to explore relationships between behaviors over many generations, with not entirely predictable results. The findings may or may not hold for particular species among mammals, and basing hypotheses on established fact and then testing them against the current balance of behaviors in particular contexts offer a stronger examination of the method; this could also involve statistical testing of findings, such as of differences between the outcomes from manipulating particular variables, and this would add robustness to findings.

Expression of pain was a feasible strategy when the energy costs of expression were low (experiment 1), when the costs of helping were lower and the benefits were higher (experiment 2), and when the environment was relatively benign in terms of lower frequency of injury (experiment 5) and injured agents might be ignored but not exploited (experiment 4). This parallels the findings that the young of various species of mammals, birds, and reptiles have distress calls which, while they risk attracting predators, are in most environments more likely to result in parental care to alleviate distress.^31^ Expression of pain was also, to a certain extent, a feasible strategy even when injury had a significant cost in time away from foraging (experiment 6). Nonexpression, or suppression of pain expression, was a better strategy when the costs of expression were high (experiment 1), and when expression rendered the agent vulnerable to exploitation (experiment 4). Other than in humans and in certain rodents, we know little about the factors affecting expression of pain in mammals, even in farm and companion animals,^47^ and even less about the responses of conspecifics.

The predominant condition that fostered helping was low-energy cost of giving help (experiment 2), consistent with findings of female mice choosing to stay close to others in pain,^28^ rat reciprocal help-giving,^18^ or herd animals grouping around an injured member.^8^ All these also favor expression of pain. However, the effects of increasing benefits of helping (rather than decreasing the costs) were rather small. It may be that even in animals that pay close attention to who helps whom^19^; the benefits of good reputation in this regard are never very substantial, or it may be that helping interacts with other factors, such as kinship or position in a social hierarchy, which we did not model. In humans, reputation and indirect reciprocation can predict helping and being helped^48^; in a study of helping in real social networks,^14^ the extent to which people were connected to others within friendship groups increased the likelihood of altruism toward the people with most connections, even when controlling for personality factors, which influence cooperativeness. For nonaltruistic behavior, or not helping, neither costs nor benefits made a noticeable difference to outcomes (experiment 2). Being nonaltruistic seemed, in general, to be a successful strategy across conditions, although this depends to an extent on the dangerousness of the environment and the likelihood of recovery without help. Furthermore, the costs of helping someone with a long-term illness or disability can be substantial, depleting both physical and mental health,^21,46^ and the tendency to loss aversion in humans (and perhaps other species) may add weight to nonaltruism as the energy-conserving default.^4^

It was evident that the effects of increasing the dangerousness of the environment, in terms of frequency of injury (experiment 5) or of losing further resources to other agents when injured and unable to defend them (experiment 4), meant that expressing pain was clearly disadvantageous. Although increasing injury frequency almost extinguished altruism, possibly because increased frequency of injury depleted the resources of altruists faster, exploitation did not; because stealing resources from the injured reduced the likelihood of their survival, demands on altruists' energy did not multiply as they did with increased injury frequency. Although the archeological record provides evidence of healed major injuries in the skeletons of humans who died of other causes,^34^ there are also contemporary and historical records of injured people being abandoned to their fate by their kin and companions in extreme adversity, as in a persecuted population in flight.^7^ It is of considerable value to deepen understanding of what conditions in human groups promote such breakdown of normal support and, at a lesser level, what conditions foster the development of exhaustion among carers.^46^

There are several obvious additions that would strengthen the model. We gave a single energy cost to expression, yet pain is expressed behaviorally in multiple ways, some visible or audible at a distance (to the extent that simulated injury may be used to draw predators from the proximity of defenseless young, best known in killdeers or plovers), whereas others such as facial expression^29,55^ are much less energy demanding and only detectable at fairly close quarters and therefore more likely (assuming the animal in pain is mobile) to be seen by conspecifics and familiars than by rivals, antagonists, or predators. Despite doubt about expression of pain in prey animals,^39^ facial expression of pain has recently been identified in horses.^15^ Furthermore, suppression of behavioral pain expression may be cognitively and emotionally costly, requiring balance against the risks of expression, and ideally, this would be modeled in further elaborations.

We did not model kinship, although it is a significant factor in whether interactions are likely to be altruistic or not,^26,50^ nor social hierarchy: both are likely to interact with the probability of helping and of being helped and by whom. In primates, where helping has been studied in relation to empathy and to theory of mind, findings may not match predictions: chimpanzees, for instance, are less likely to help kin than nonkin, despite help-giving being costly.^41^ Agents could be allowed to learn from injury such that they became less likely over their life spans to be reinjured; injury severity could vary within trials, not only between trials.

Importantly, agents could be enabled to discriminate among those they interacted with, offering help to those who had helped them, and ignoring those who had ignored them when they were injured. Reputation, and even being observed by others, can have powerful effects on behavior.^27^ Although the difference between one-off and repeated interactions can be crucial to behavioral decisions,^17^ the dynamics of repeated interactions may also determine behavior in one-off interactions because it is rarely certain that any interaction could not be repeated. Selective helping according to interaction history may not be limited to primates; it may also be apparent in some rodents.^18^

This study has shown, however, that the costs of showing pain and responding to others' pain may be offset by benefits to both agents in the interaction and that, while the behaviors are sensitive to conditions in the environment, the behaviors do not necessarily disappear in any but the most benign environments. In humans, altruism and helping is often investigated through economic games,^21^ of questionable generalizability to actual helping,^14^ such as in situations based on shared experience of need or pain. Responses to others' pain are not universally helpful; they can be neutral, uncaring, or actively cruel,^23,56^ both in informal and in institutional settings. Understanding the dynamics of expressing or suppressing pain experience and the dynamics of choice between responses, as elaborated by Williams,^55^ is of importance far beyond the clinical setting.

We have remarkably few data on animals' spontaneous behavior in relation to pain; Patrick Wall^52^ wrote one of the few accounts. There are even fewer observations of interactions between conspecifics, 1 or more of which is displaying pain. As a method of simulating possible interactions and their outcomes, agent-based modeling offers possibilities of refining hypotheses to be tested in vivo, whether by experimental manipulation or by systematic observation and tracking in the wild. The method is a promising one for investigating wider questions about behaviors associated with pain^57^ whether behaviors have specific functions in different phases of threat, injury, and recovery,^13,53^ the possible functions of different pain behaviors and the utility of classifications such as automatic vs controlled^25^ or protective vs communicative^44^; the environmental and social variables that may affect animal behavior relevant to animal experiments on pain and analgesia.^43^

## Conflict of interest statement

The authors have no conflicts of interest to declare.

Some of this work was supported by funding from the Engineering and Physical Sciences Research Council (EPSRC) awarded to E. Gallagher through UCL CoMPLEX.

## Supplementary Material

SUPPLEMENTARY MATERIAL

## Appendix A Supplemental Digital Content

Supplemental Digital Content associated with this article can be found online at http://links.lww.com/PAIN/A191.

## References

[1] Ashley PJ Ringrose S Edwards KL Wallington E McCrohan CR Sneddon LU Effect of noxious stimulation upon antipredator responses and dominance status in rainbow trout. Anim Behav 2009;77:403–10.

[2] Axelrod RM The complexity of cooperation: Agent-based models of competition and collaboration. Princeton, New Jersey: Princeton University Press, 1997.

[3] Axelrod R Dion D The further evolution of cooperation. Science 1988;242:1385–90. 17802133 10.1126/science.242.4884.1385

[4] Baumeister RF Bratslavsky E Finkenauer C Vohs KD Bad is stronger than good. Rev Gen Psychol 2001;5:323–70.

[5] Ben-Ami Bartal I Decety J Mason P Empathy and pro-social behaviour in rats. Science 2011;334:1427–30. 22158823 10.1126/science.1210789 PMC3760221

[6] Black H Underwater suffering? Scientific Am Mind 2009;20:16.

[7] Black M Collapsing health care in Serbia and Montenegro. BMJ 1993;307:1135–7. 8251816 10.1136/bmj.307.6912.1135 PMC1679149

[8] Bode NWF Faria JJ Franks DW Krause J Wood AJ How perceived threat increases synchronization in collectively moving animal groups. Proc Biolog Sci B 2010;277:3065–70. 10.1098/rspb.2010.0855 PMC2982070 20504810

[9] Brown JL Sheffield D Leary MR Robinson ME Social support and experimental pain. Psychosom Med 2003;65:276–83. 12651995 10.1097/01.psy.0000030388.62434.46

[10] Carere C Grignani G Bonanni R Della Gala M Carlini A Angeletti D Cimmaruta R Nascetti G Mather JA Consistent individual differences in the behavioural responsiveness of adult male cuttlefish (Sepia officinalis). Appl Anim Behav Sci 2015;167:89–95.

[11] Coan JA Schaefer HS Davidson RJ Lending a hand: social regulation of the neural response to threat. Psychol Sci 2006;17:1032–9. 17201784 10.1111/j.1467-9280.2006.01832.x

[12] Cosmides L Tooby J Cognitive adaptations for social exchange. In: Barkow JH Cosmides L Tooby J, editors. The adapted mind. Oxford, England: Oxford University Press, 1992 p. 163–228.

[13] Crook RJ Hanlon RT Walters ET Squid have nociceptors that display widespread long-term sensitization and spontaneous activity after bodily injury. J Neurosci 2013;33:10021–6. 23761897 10.1523/JNEUROSCI.0646-13.2013 PMC4767780

[14] Curry O Dunbar RIM Altruism in networks: the effect of connections. Biol Lett 2011;7:651–3. 21411451 10.1098/rsbl.2010.1202 PMC3169038

[15] Dalla Costa E Minero M Lebelt D Stucke D Canali E Leach MC Development of the Horse Grimace Scale (HGS) as a pain assessment tool in horses undergoing routine castration. PLoS One 2014;9:e92281. 24647606 10.1371/journal.pone.0092281 PMC3960217

[16] Decety J Norman GJ Berntson GG Cacioppo JT A neurobehavioral evolutionary perspective on the mechanisms underlying empathy. Progr Neurobiol 2012;98:38–48. 10.1016/j.pneurobio.2012.05.001 22580447

[17] Delton AW Krasnow MM Cosmides L Tooby J Evolution of direct reciprocity under uncertainty can explain human generosity in one-shot encounters. Proc Nat Academ Sci 2011;108:13335–40. 10.1073/pnas.1102131108 PMC3156224 21788489

[18] Dolivo V Taborsky M Norway rats reciprocate help according to the quality of help they received. Biol Lett 2014;11:0959. 10.1098/rsbl.2014.0959 PMC4360107 25716088

[19] Dugatkin LA Cooperation among animals. Oxford, England: Oxford University Press, 1997.

[20] Farmer JD Foley D The economy needs agent-based modelling. Nature 2009;460:685–6. 19661896 10.1038/460685a

[21] Fehr E Fischbacher U The nature of human altruism. Nature 2003;425:785–91. 14574401 10.1038/nature02043

[22] Frank RH Passions within reason: the strategic role of the emotions. New York: WW Norton & Co., 1988.

[23] Goubert L Craig KD Vervoort T Morley S Sullivan MJL Williams A Cano A Crombez G Topical review: Facing others in pain: the effects of empathy. PAIN 2005;118:285–8. 16289804 10.1016/j.pain.2005.10.025

[24] Gould SJ Lewontin RC The spandrels of San Marco and the Panglossian program: a critique of the adaptationist programme. Proc R Soc Lond 1979;205:281–8. 10.1098/rspb.1979.0086 42062

[25] Hadjistavropoulos T Craig KD A theoretical framework for understanding selfreport and observational measures of pain: a communications model. Behav Res Ther 2010;40:551–70. 12038648 10.1016/s0005-7967(01)00072-9

[26] Hamilton WD Selection of selfish and altruistic behaviour in some extreme models. In: Hamilton WD, editor. Narrow roads to gene land, Vol. 1 Oxford, England: Macmillan Press, 1971/1996 p. 185–227.

[27] Izuma K. The social neuroscience of reputation. Neurosci Res 2012;72:283–8. 22285602 10.1016/j.neures.2012.01.003

[28] Langford DJ Crager SE Shehzad Z Smith SB Sotocinal SG Levenstadt JS Chanda ML Levitin DJ Mogil JS Social modulation of pain as evidence for empathy in mice. Science 2006;312:1967–70. 16809545 10.1126/science.1128322

[29] Langford DJ Bailey AL Chanda ML Clarke SE Drummond TE Echols S Glick S Ingrao J Klassen-Ross T Lacroix-Fralish ML Matsumiya L Sorge RE Sotocinal SG Tabaka JM Wong D van den Maagdenberg AMJM Ferrari MD Craig KD Mogil JS Coding of facial expressions of pain in the laboratory mouse. Nat Methods 2010;7:447–9. 20453868 10.1038/nmeth.1455

[30] Langford DJ Tuttle AH Briscoe C Harvey-Lewis C Baran I Gleeson P Fischer DB Buonora M Sternberg WF Mogil JS Varying perceived social threat modulates pain behavior in male mice. J Pain 2011;12:125–32. 20685172 10.1016/j.jpain.2010.06.003

[31] Lind J Cresswell W Determining the fitness consequences of antipredation behavior. Behav Ecol 2005;16:945–56.

[32] Macal CM North MJ Tutorial on agent-based modeling and simulation part 2: how to model with agents. Perrone LF Lawson BG Liu J Wieland FP(Eds). In: Proceedings of the 38th Winter Simulation Conference, Monterey, CA, 2006 p. 73–83.

[33] Magee B Elwood RW Shock avoidance by discrimination learning in the shore crab (Carcinus maenas) is consistent with a key criterion for pain. J Exper Biol 2013;216:353–8. 23325857 10.1242/jeb.072041

[34] Mithen S The prehistory of the mind. London, England: Thames & Hudson, 1996.

[35] Mogil JS Animal models of pain: progress and challenges. Nat Rev Neurosci 2009;10:283–94. 19259101 10.1038/nrn2606

[36] Mogil JS The surprising empathic abilities of rodents. Trends Cog Sci 2012;16:143–4. 10.1016/j.tics.2011.12.012 22206750

[37] Mogil JS Social modulation by and of pain in humans and rodents. PAIN 2015;156:S35–S41. 25789435 10.1097/01.j.pain.0000460341.62094.77

[38] Niazi M Hussain A Agent-based computing from multi-agent systems to agent-based models: a visual survey. Scientometrics 2011;89:479–99.

[39] Nolan A Do animals feel pain like we do? The Conversation 7th July, 2015. Available at: http://theconversation.com/do-animals-feel-pain-like-we-do-42508. Accessed 25th September, 2015.

[40] Nowak MA Sigmund K Evolution of indirect reciprocity. Nature 2005;437:1291–8. 16251955 10.1038/nature04131

[41] O'Connell S Empathy in chimpanzees: evidence for theory of mind? Primates 1995;36:397–410.

[42] Peeters PA Vlaeyen JW Feeling more pain, yet showing less: the influence of social threat on pain. J Pain 2011;12:1255–61. 22050970 10.1016/j.jpain.2011.07.007

[43] Price TJ Dussor G Evolution: the advantage of “maladaptive” pain plasticity. Curr Biol 2014;24:R384–7. 24845663 10.1016/j.cub.2014.04.011 PMC4295114

[44] Prkachin KM Pain behaviour is not unitary. Beh Brain Sci 1986;9:754–5.

[45] Smith ES Lewin GR Nociceptors: a phylogenetic view. J Comp Physiol 2009;195:1089–106. 19830434 10.1007/s00359-009-0482-z PMC2780683

[46] Smith L Onwumere J Craig T McManus S Bebbington P Kuipers E Mental and physical illness in caregivers: results from an English national survey sample. Br J Psychiatry 2014;205:197–203. 25061119 10.1192/bjp.bp.112.125369

[47] Sneddon LU Defining and assessing animal pain. Anim Behav 2014;97:201–12.

[48] Sperber D Baumard N Moral reputation: an evolutionary and cognitive perspective. Scholarly Commons Available at: http://repository.upenn.edu/goldstone/8 dated 10–29–2012. Accessed 10th January, 2015.

[49] Tooby J Cosmides L Friendship and the banker's paradox: other pathways to the evolution of adaptations for altruism. Proc Br Acad 1996;88:119–43.

[50] Trivers RL The evolution of reciprocal altruism. Quart Rev Biol 1971;46:35–57.

[51] Vlaeyen JWS Hanssen M Goubert L Vervoort T Peters M van Breukelen G Sullivan MJL Morley SJ Threat of pain influences social context effects on verbal pain report and facial expression. Behav Res Ther 2009;47:774–782. 19570524 10.1016/j.brat.2009.05.008

[52] Wall PD On the relation of injury to pain. PAIN 1979;6:253–64. 460933 10.1016/0304-3959(79)90047-2

[53] Walters ET Injury-related behavior and neuronal plasticity: an evolutionary perspective on sensitization, hyperalgesia and analgesia. Int Rev Neurobiol 1994;36:325–427. 7822120 10.1016/s0074-7742(08)60307-4

[54] Wiech K Tracey I Pain, decisions, and actions: a motivational perspective. Frontiers Neurosci 2013;7:46. 10.3389/fnins.2013.00046 PMC3613600 23565073

[55] Williams ACdeC Facial expression of pain: an evolutionary account. Behav Brain Sci 2002;25:439–55. 12879700 10.1017/s0140525x02000080

[56] Williams ACdeC Craig KD A science of pain expression? PAIN 2006;125:202–3. 16962239 10.1016/j.pain.2006.08.004

[57] Williams ACdeC What can evolutionary theory tell us about chronic pain? PAIN. 10.1097/j.pain.0000000000000464 [ePub ahead of print]. 10.1097/j.pain.0000000000000464 26683235

[58] Williams GC Adaptation and natural selection. Princeton, NJ: Princeton University Press, 1996.

