# Supplementary material for: Pain expressiveness and altruistic behavior: an exploration using agent-based modeling
Source: Pain. 2015 Nov 26;157(3):759–68. doi: 10.1097/j.pain.0000000000000443 (PMC4751745; doi:10.1097/j.pain.0000000000000443)
Supplement: SUPPLEMENTARY MATERIAL [file jop-157-759-s001.docx]

**Supplemental data 1 MATLAB code**

**Finding the initial agent population**

1 function agents = ...

initialagents(numberagents,minc,maxc,maxage,perexandalt,perexandself,...

2 pernonexandalt,pernonexandself,initenergy)

3

4 % INPUT:

5

6 % numberagents: Number of agents wanted

7 % minc: Minimum connectedness score

8 % maxc: Maximum connectedness score

9 % maxage: Maximum age possible

10 % perexandalt: Initial proportions of altruistic expressers

11 % perexandself: Initial proportions of selfish expressers

12 % pernonexandalt: Initial proportions of altruistic non−expressers

13 % pernonexandself: Initial proportions of selfish non−expressers

14 % initenergy: Initial energy of agents

15

16 17 18 19 20 21 22 23 24 25 26 27 28 29 30 31

32

33 34 35 36 37 38 39

40

41

%% ======== CONNECTEDNESS =================================================

% Generate 100 random elements from an exponential distribution with mean 1

meancon = 1;

alldist = exprnd(meancon,numberagents,1);

% Normalise to be between maxc and minc:

maxd = max(alldist);

normalised = (alldist/maxd)*(maxc−minc);

% Connectedness values for initial agent population:

con = normalised + minc;

%% ======== AGE ===========================================================

% Each agent can be an age between 0 and 100, we will start with a random

% distribution of these ages in our initial population

ages = round(rand(numberagents,1)*(maxage−1)); % so it can't be maxage ... when it starts

%% ======== INTIAL STRATEGIES =============================================

% First column: 1 if an expresser, 0 if a non−expresser

% Second column: 1 if an altruist, 0 if selfish

e1 = [ones(perexandalt*numberagents,2)];

e2 = [ones(perexandself*numberagents,1),zeros(perexandself*numberagents,1)];

e3 = ...

[zeros(pernonexandalt*numberagents,1),ones(pernonexandalt*numberagents,1)];

e4 = [zeros(pernonexandself*numberagents,2)];

42

43 44 45 46

47

48

ae = [e1;e2;e3;e4];

rae = ae(randperm(length(ae)),:);

%% ======== INITIAL AGENT POPULATION ======================================

agents = [rae(:,1) rae(:,2) con initenergy*ones(numberagents,1) ages ...

zeros(numberagents,1) (1:numberagents)'];

end

**Implementing the agent-based model**

1 function [totstrats distconn disten meanenergystrats numdied numberinpain ...

numberofinteractions agesoftheoneswhowilldie] = ...

2 painabm(totaliterations,maxc, minc,numberagents,initenergy,...

3 maxenergy,maxage,ageinc,forageinc,timepain,numinjured,inheritcon,...

4 cexp,calt,cself,balt,agents,interactionscaling)

5

6 % INPUT:

7 % totaliterations: Number of iterations

8 % maxc: Maximum connectedness

9 % minc: Minimum connectedness

10 % numberagents: Number of agents

11 % initenergy: Initial energy that all agents have

12 % maxenergy: Maximum energy that an agent can have

13 % maxage: Maximum age before an agent dies

14 % ageinc: How much an agent ages each iteration

15 % forageinc: How much energy received from foraging each iteration

16 % timepain: Maximum number of iterations in pain (if not helped)

17 % numinjured: Number of agents put into pain each iteration, n

18 % inheritcon: 1 or 0, whether you should inherit connectedness,

19 % or 0: just have a random connectedness

20 % cexp: Energy cost of expressing

21 % calt: Energy cost of helping an agent in pain

22 % cself: Connectedness cost of being selfish

23 % balt: Connectedness benefit of being altruistic

24 % agents: The initial population of agents

25 % interactionscaling: Sociability shift, s

26 % how much an agent's connectedness score is shifted when

27 % finding its chance of interacting.

28 %

29 %

30 % OUTPUT:

31 % totstrats: The number of agents using different strategies at each

32 % iteration

33 % distconn: Distribution of connectedness each iteration

34 % disten: Distribution of energy levels each iteration

35 % meanenergystrats: Mean energy of agents using different strategies each

36 % iteration

37 % numdied: Total agents who got replaced each iteration

38 % numberofinteractions:

39 % Number of interactions there were each iteration

40 % numberinpain: Total number in pain each iteration

41 % agesoftheoneswhowilldie: Ages of the ones who have died each iteration.

42

43 bexp = timepain; % Benefit of expressing pain is that your time in pain ...

goes to zero.

44 meancon = 1; % The mean of the exponential distribution used to get ...

45

46 47

connectedness values.

%% AN AGENT:

% [expresser?(1/0), altruistic?(1/0), connectedness score, energy, age, ...

time−out, index number];

48

49 50 51 52 53 54 55 56 57 58 59 60 61 62 63 64 65 66

67

68 69 70 71 72 73 74 75 76 77 78 79 80

81

82 83 84 85 86 87 88 89 90 91 92 93 94 95 96 97 98 99

100

for t = 1:totaliterations

ttt = 1; % Ages of the ones which will die counter

%% ======== CHOOSING AGENTS FOR INJURY ================================

% Which agents will be selected for pain this iteration?

% 'numinjured' random agentS get injured (who are not already in pain)

if sum(agents(:,6)==0)=0 % If there are healthy agents

if numinjured*>*size(agents(:,6)==0,1)

% If there are more chosen for injury than agents who are

% healthy, just injur all the remaining healthy agents

inpain = agents(:,7);

else

% Pick 'numinjured' lots of random agents for injury

inpain = datasample(agents(agents(:,6)==0,7),numinjured);

end

% For these/this chosen agent the time in pain is set from 0 to ...

"timepain"

agents(inpain,6) = timepain;

end

clear inpain

%% ======== CHOOSING INJURED AGENTS FOR INTERACTION ===================

% Being interacted with is based on connectedness scores

% The agents in pain:

theagentsinpain = agents(agents(:,6)=0,:);

if size(theagentsinpain,1)==1

% For the agents in pain, if there are any, find out if they get ...

an interaction or

% not based on their connectedness score

ischosenint = zeros(size(theagentsinpain,1),1);

for i = 1:size(theagentsinpain,1)

% Include sociability shift

bb = theagentsinpain(i,3)+interactionscaling;

if bb*>*maxc

% If an agent has this shifted score *>* maxc, set it to maxc

bb = maxc;

end

% Find whether it interacts or not

if rand(1,1)*<*bb

ischosenint(i) = theagentsinpain(i,7);

end

clear bb

end

% The indexes of the agents chosen for interaction:

chosenint = ischosenint(ischosenint=0);

end

%% ======== BEGIN INTERACTIONS =====================================

101

102 103 104 105

106

107 108

109

110 111 112 113 114 115 116 117 118 119 120 121

122

123

% If there are interactions this iteration

if exist('chosenint')==1

ni = length(chosenint); % The number of agents being interacted with

% for these chosen agents in pain, let them interact with any ...

agent randomly

hchosenint = zeros(ni,1); % Preallocation for pairing agents

for eachchosen = 1:ni

hchosenint(eachchosen) = ...

randsample(agents(agents(:,7)=chosenint(eachchosen)...

&ismember(agents(:,7),hchosenint),7),1);

end

% Interaction pairs

if size(chosenint,2)=1

intpairs = [chosenint' hchosenint];

else

intpairs = [chosenint hchosenint];

end

% COSTS AND BENEFITS OF THE INTERACTIONS:

for i = 1:ni

if (agents(intpairs(i,1),1)==1)&&(agents(intpairs(i,2),2)==1) ...

% expresser vs altruistic

% AGENT IN PAIN, EXPRESSING:

124

125

126

127

128

129

130 agents(intpairs(i,1),4) = agents(intpairs(i,1),4) − cexp; % Energy cost of expressing

agents(intpairs(i,1),6) = agents(intpairs(i,1),6) − bexp; % Less iterations left in pain

% HEALTHY AGENT WHO IS INTERACTING, ALTRUISTIC:

agents(intpairs(i,2),4) = agents(intpairs(i,2),4) − calt; % Energy cost of helping

agents(intpairs(i,2),3) = agents(intpairs(i,2),3) + balt; % Connectedness increases

elseif …

131

132 133

134

135 136

137

138 139 140

141

142 143

end

else

end

(agents(intpairs(i,1),1)==1)&&(agents(intpairs(i,2),2)==0) ...

% expresser vs selfish

% AGENT IN PAIN, EXPRESSING:

agents(intpairs(i,1),4) = agents(intpairs(i,1),4) − cexp; % Energy cost of expressing

% Subtract the energy stolen here if using antagonists

% HEALTHY AGENT WHO IS INTERACTING, SELFISH:

agents(intpairs(i,2),3) = agents(intpairs(i,2),3) − cself; % Connectedness decreases

% Add the energy stolen here if using antagonists

% In the cases of non−expresser vs altruistic and ...

non−expresser vs selfish

% there are no costs or benefits

144

145 146 147 148 149 150 151 152 153 154 155 156 157 158 159 160 161 162 163 164 165 166 167 168 169 170 171 172 173 174 175 176 177 178 179 180 181 182 183 184 185 186 187 188 189 190 191 192 193 194 195 196 197 198

end

%% ======== AGING, FORAGING AND PAIN RECOVERY====================

% Let the agents age

agents(:,5) = agents(:,5) + ageinc;

% For the ones not in pain let them forage

notinpainagents = agents(agents(:,6)==0,7);

agents(notinpainagents,4) = agents(notinpainagents,4) + forageinc;

% If an agent in pain has not been helped in this iteration, decrease

% it's time left in pain by 1

for i = 1:numberagents

if agents(i,6)=0

agents(i,6) = agents(i,6)−1;

end

end

%% ======== MAKE SURE LEVELS ARE WITHIN LIMITS ========================

% Connectedness:

agents(agents(:,3)*>*maxc,3) = maxc;

agents(agents(:,3)*<*minc,3) = minc;

% Iterations left in pain:

agents(agents(:,6)*>*timepain,6) = timepain;

agents(agents(:,6)*<*0,6) = 0;

% Energy:

agents(agents(:,4)*>*maxenergy,4) = maxenergy;

agents(agents(:,4)*<*0,4) = 0;

% Age:

agents(agents(:,5)*>*maxage,5) = maxage;

%% ======== DEATH/BIRTH ===============================================

% Which agents should die now? death for: energy = 0 and age = maxage

j=1;

for i = 1:numberagents

if agents(i,4) = = 0   agents(i,5)==maxage; % The agents who should die.

todelete(j) = i; % Indexes of those who will be deleted.

agesoftheoneswhowilldie(t,ttt) = agents(i,5); % What is their age?

ttt = ttt+1;

j = j+1;

end

end

if exist('todelete') % If there are agents due to die

%% ======== SELECTING PARENTS =====================================

% Agents not about to be deleted:

199

200

201 202 203 204 205 206 207 208 209 210 211 212 213 214 215 216 217 218 219 220 221 222

223

224

225

226 227 228 229 230 231 232 233 234 235

236

237 238 239 240 241 242

healthyparents = agents(ismember(agents(:,7),todelete),:); % All ...

the details of these agents

% Parents chosen weighted on their energy score

xp = healthyparents;

m = randn(1,1000);

mm = (1−abs(m)/max(abs(m)))*maxenergy;

% A normal distribution with values between 0 and 100

for i = 1:length(todelete)*2 % 2 parents per new agent

% Normalise the possible parents' energy scores:

normhealthyparents = [xp(:,4)./(max(xp(:,4))) xp(:,7)];

% Which of these possible parents' energy scores is closest to

% a number selected from mm

[cp indexp] = min(abs(normhealthyparents(:,1)−mm(i)));

% This agent will be chosen for parenthood

% If there is more than one with this same closest energy

% score, then pick randomly between them

if sum(abs(normhealthyparents(:,1)−mm(i))==cp)=1

% If there is more than one agent with this same energy

rp = sum(abs(normhealthyparents(:,1)−mm(i))==cp); % Number ...

that are the same

rrp = randsample(1:rp,1); % Pick one randomly rrrp = ...

normhealthyparents(abs(normhealthyparents(:,1)−mm(i))==cp,2);

% Select this agent to be a parent

chosenparent(i) = rrrp(rrp);

% Take this agent out of the list, to select another:

xp = xp(xp(:,7)=rrrp(rrp),:);

else

% If there is only one agent with this energy

% select this agent for parenthood:

chosenparent(i) = normhealthyparents(indexp,2); % This is ...

added to the list of agents to interact

% Take this agent out of the list, to select another:

xp = xp(xp(:,7)=normhealthyparents(indexp,2),:);

end

clear normhealthyparents cp indexp

end

243 %% ======== AGENT REPLACEMENT ====================================

244

245 246 247 248 249

% Replace 'todelete' dying agents with an offspring agent from the

% selected parents

if exist('chosenparent')==1 % If there are any dying agents

250

251 252 253 254 255 256 257 258 259 260 261 262 263 264

265

266

267

268 269 270 271 272 273 274 275 276 277 278 279 280 281 282 283

284

285

286 287

end

% Parent pairs

parents = reshape(chosenparent,length(todelete),2);

% Distribution to take connectedness value from if inheritcon =

%0

babyalldist = exprnd(meancon,1000,1);

babymaxd = max(babyalldist);

babynormalised = (babyalldist/babymaxd)*(maxc−minc);

babyconnectedness = babynormalised+minc;

for i = 1:length(todelete)

% Connectedness

if inheritcon == 1 % Inherit connectedness

babycon = ...

mean([agents(parents(i,1),3),agents(parents(i,2),3)]);

else

babycon = babyconnectedness(i); % or select from ...

exponential dist

end

% Expression

if agents(parents(i,1),1)=agents(parents(i,2),1)

babyexp = round(rand(1));

else

babyexp = agents(parents(i,1),1);

end

% Altruism

if agents(parents(i,1),2)=agents(parents(i,2),2)

babyalt = round(rand(1));

else

babyalt = agents(parents(i,1),2);

end

% Replace the old agent with the new one, same index as ...

the old one.

agents(todelete(i),:) = [babyexp babyalt babycon ...

initenergy 0 0 todelete(i)]; % New agent

clear babycon babyexp babyalt

end

288 clear babyalldist babymaxd babynormalised babyconnectedness

289 end

290

291 292 293 294 295 296 297 298 299 300

%% ======== VARIABLES TO OUTPUT =======================================

% The number of agents using different strategies there are:

totstrats(1,t) = size(agents((agents(:,1)==1)&(agents(:,2)==1),:),1);

totstrats(2,t) = size(agents((agents(:,1)==1)&(agents(:,2)==0),:),1);

totstrats(3,t) = size(agents((agents(:,1)==0)&(agents(:,2)==1),:),1);

totstrats(4,t) = size(agents((agents(:,1)==0)&(agents(:,2)==0),:),1);

if size(agents,1)==numberagents

distconn(t,1:numberagents) = agents(:,3);

301

302 303 304 305 306

else

end

disten(t,1:numberagents) = agents(:,4);

distconn(t,1:numberagents) = zeros(1,numberagents);

disten(t,1:numberagents) = zeros(1,numberagents);

307 308 309 310 311 312 313 314 315 316 317 318 319 320 321

322

323 324 325 326 327 328

329

330

331

end end % Mean energy for each strategy

meanenergystrats(1,t) = mean(agents(agents(:,1)==1&agents(:,2)==1,4));

meanenergystrats(2,t) = mean(agents(agents(:,1)==1&agents(:,2)==0,4));

meanenergystrats(3,t) = mean(agents(agents(:,1)==0&agents(:,2)==1,4));

meanenergystrats(4,t) = mean(agents(agents(:,1)==0&agents(:,2)==0,4));

if exist('todelete')

numdied(t) = length(todelete); % Total agents who got replaced

else

numdied(t) = 0;

end

if exist('ni')

numberofinteractions(t) = ni; % Number of interactions there were ...

this iteration

else

numberofinteractions(t) = 0;

end

numberinpain(t) = size(agents(agents(:,6)=0),1);

clear ww numhealthy numinterations numpain yy yyy agentsinpain x ...

chosenint ...

ni intpairs todelete healthyparents xp m mm chosenparent parents ...

theagentsinpain ischosen
