# Supplementary material for: Pain expressiveness and altruistic behavior: an exploration using agent-based modeling
Source: Pain. 2015 Nov 26;157(3):759–68. doi: 10.1097/j.pain.0000000000000443 (PMC4751745; doi:10.1097/j.pain.0000000000000443)

**Supplemental data 2 Preliminary simulations and calibration**

For simplicity, the energy cost of expressing pain and the energy cost of helping were set to the energy increment from foraging: *c_exp_* = *c_alt_* = 1. An agent’s connectedness score determined its changes of interacting. For initial runs, 1% of agents were injured at each iteration, and other cost and benefit values were taken as their mid-values.

Then the model was run with low, mid, and high values for *t_p_*_,_ *c_self_*, and *b_alt_*, with the results seen in Figure S1 below. From these, it is clear that little impact on strategy outcomes arises from *c_self_* or *b_alt_*, so a mid-value was used.

Increasing *t_p_* had the effect of increasing the proportion of expressive altruists, in what appeared to be a linear relationship. Using a mid-value for *t_p_* produced a reasonable number of agents in pain at each iteration (as the effect of injury carried over multiple iterations until recovery was complete).

Figure S1 Proportion of strategies in the population at each iteration where time in pain *t_p_*, connectedness benefit of altruism *b_alt_* and connectedness cost of selfishness *c_sel_*_f_ are varied between low, mid and high values (as shown above each). Initial settings *c_exp_* = *c_alt_* = 1. 3000 iterations averaged over 10 trials.


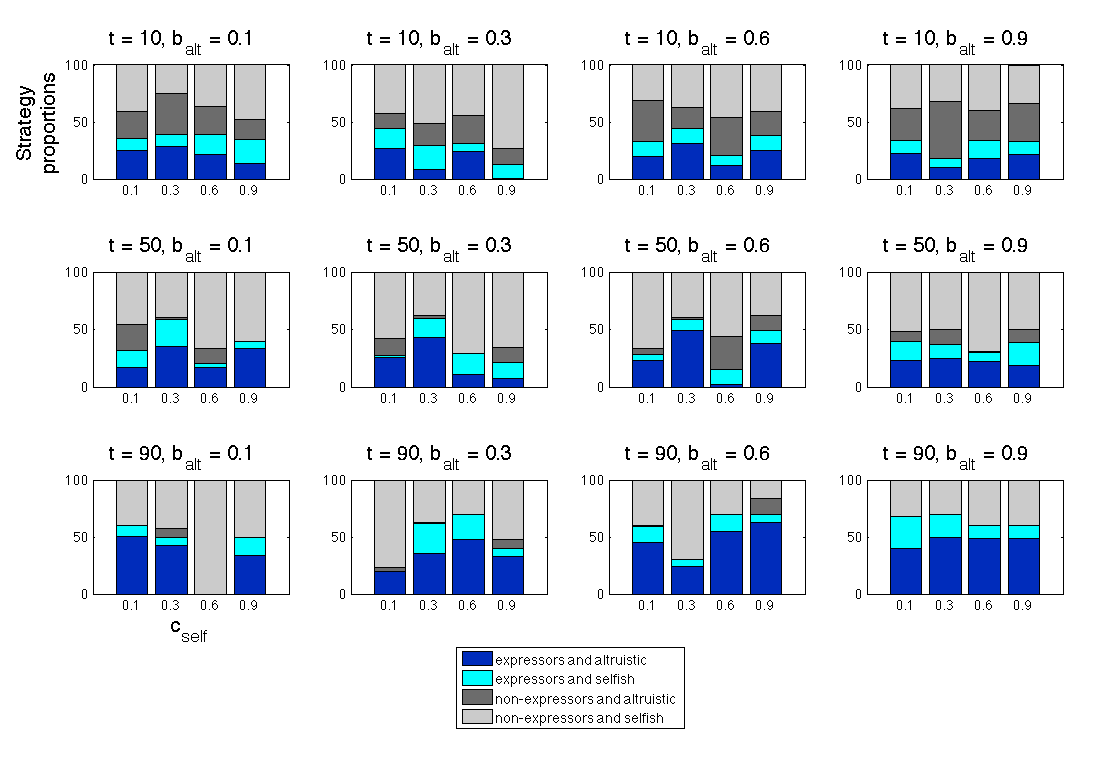


Figure S2: The final proportions of strategies in the population run 10 times using the same default initial agent population (a) and run using 10 other initial populations (b). Default parameters were used and results averaged over 100 repeated simulations run for 10,000 iterations.

1. (b)


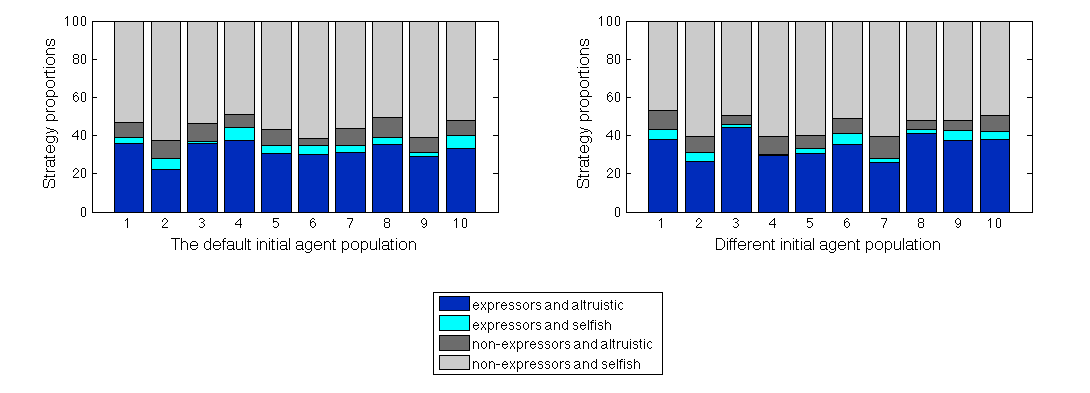

Supplement: SUPPLEMENTARY MATERIAL [file jop-157-759-s002.docx]
