# Supplementary material for: Pain expressiveness and altruistic behavior: an exploration using agent-based modeling
Source: Pain. 2015 Nov 26;157(3):759–68. doi: 10.1097/j.pain.0000000000000443 (PMC4751745; doi:10.1097/j.pain.0000000000000443)

**Supplemental data 3**

Figure S3 Additional runs to those in Figure 2 in main text, showing eventual balance of strategies


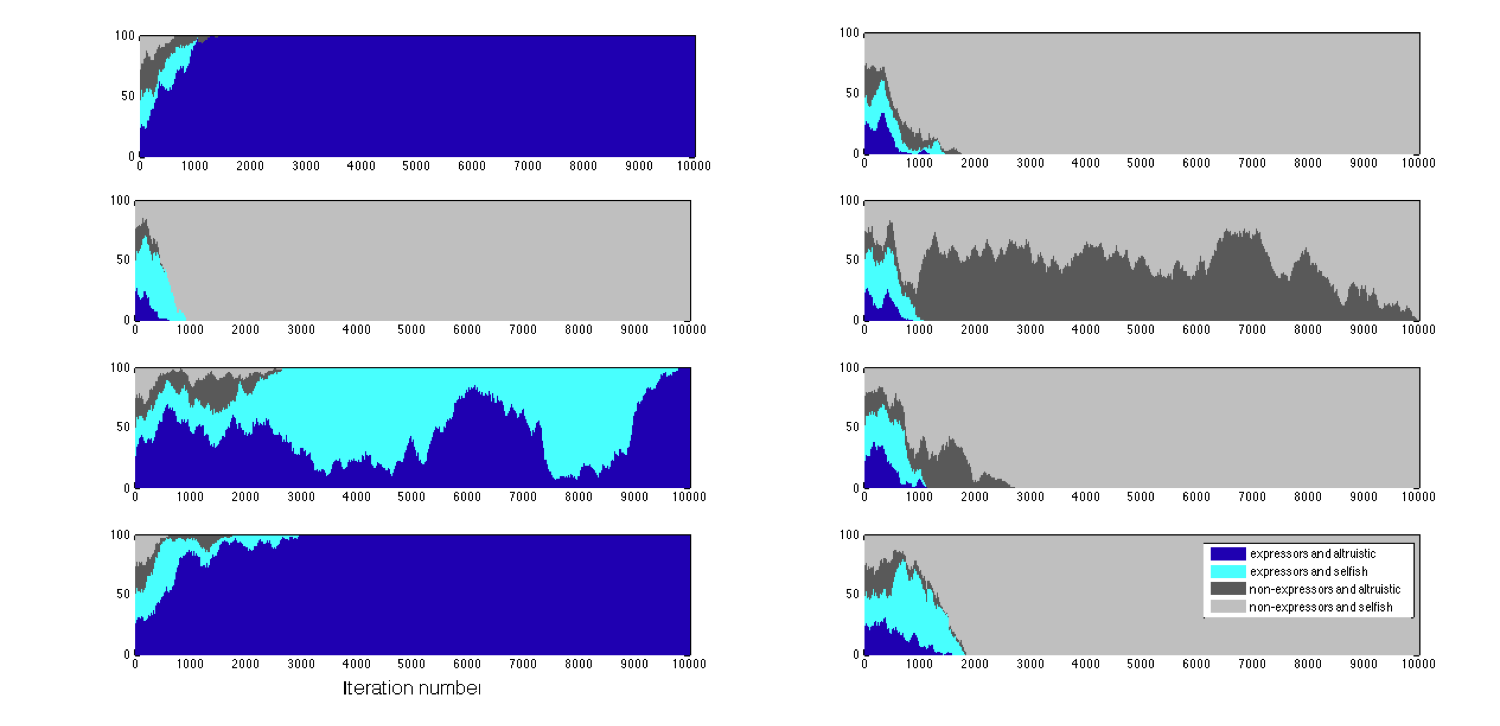

Supplement: SUPPLEMENTARY MATERIAL [file jop-157-759-s003.docx]
